# Linking Visual–Auditory Cues to Restoration: The Mediating Role of Perceived Biodiversity

**DOI:** 10.3390/ijerph22081267

**Published:** 2025-08-13

**Authors:** Jaeyoung Ha, Hyung Jin Kim, M M Lekhon Alam

**Affiliations:** 1 Landscape Architecture Program, Virginia Tech, Blacksburg, VA 24061, USA; 2 Department of Landscape Architecture and Regional & Community Planning, Kansas State University, Manhattan, KS 66506, USA; hyungjin@ksu.edu; 3 Department of Technology Systems, East Carolina University, Greenville, NC 27858, USA; alamm22@ecu.edu

**Keywords:** restorative environments, urban green space, auditory–visual stimuli, preference, urban landscapes

## Abstract

Due to rapid urbanization over the past five decades, there has been growing interest in the role of biodiversity in supporting human well-being. While previous research highlights the role of landscape biodiversity in psychological restoration, the pathway linking visual and auditory cues to perceived biodiversity—and subsequently to restorative outcomes—remains poorly understood. This study explores how visual and auditory stimuli influence human perception, including perceived biodiversity, preference, and restorative effects, within environments that maintain a consistent level of ecological biodiversity. This study constructed 16 hypothetical environments by combining three visual factors (species evenness, vegetation height, and plant color) with one auditory factor (presence or absence of natural sound), holding actual biodiversity constant. By comparing results from ANOVA and mixed-effect modeling, our analysis revealed important contrasts between the direct and indirect effects of visual and auditory features on perceived biodiversity and restoration. Plant height and natural sound consistently demonstrated direct positive effects on restorative outcomes. In contrast, plant color and species evenness influenced restoration indirectly, mediated through perceived biodiversity. The mixed-effect model indicated a partial mediation pathway between landscape features and restorative effects—an effect not observed in the ANOVA analysis. Surprisingly, species evenness was not directly associated with restorative outcomes, but was indirectly linked via perceived biodiversity. Similarly, while color enhanced biodiversity perception, it did not directly improve mental restoration and, under some conditions, may even contribute to overstimulation. These findings suggest that the restorative benefits of nature arise not only from the ecological composition of landscapes but also from how biodiversity is perceived. Designers and planners should consider not only biodiversity itself, but also how it is presented and perceived through multisensory experiences.

## 1. Introduction

In recent decades, scholarship has investigated how urban green space can contribute to restorative benefits for individuals. Restorative environments are natural settings that facilitate recovery from mental fatigue in an effortless manner [1]. In a wider context, restorative benefits include mental, emotional, cognitive, and physiological well-being. Numerous studies have found that the quantity of urban green space plays a pivotal role in the amelioration of mental health problems through stress reduction [2], social interactions [3], physical activities [4], preference [5], and thermal comfort [6]. In other words, the more green space, the greater the restorative benefits. However, few studies have examined what characteristics of urban green space maximize the benefits of nature for human well-being beyond its quantity [7,8].

A nascent yet expanding body of research on restorative landscape investigates the relationship between urban green space biodiversity and mental well-being. There is growing evidence for the positive relationship between species richness and restorative benefits [9,10,11,12]. Green spaces with high biodiversity promote a sense of soft fascination and beauty, allowing people to fall into a state of involuntary attention [1,13,14] Furthermore, greater biodiversity environments lead to a reduction in brain activity, facilitating relaxation and attention compared to lower-biodiversity environments [15]. According to Kaplan and Kaplan’s preference matrix landscape visual complexity (e.g., diversity, richness) determines the preference of landscape scenes [1], indicating the main factor of restorative potential [16]. Along with visual perception, a biodiverse environment can provide people with natural sounds from bird and insect songs, which also deepens the restoration effect [11].

Though biodiversity appears to be a crucial indicator of mental well-being, it is perceived biodiversity—the degree to which individuals subjectively interpret the richness of species—that may play a more pivotal role in affecting mental restoration than the actual (objective) level of biodiversity. Thus, understanding how people perceive biodiversity is critical for designing restorative environments that enhance cognitive and psychological experiences. Research such as Schebella et al.’s study shows that perceived biodiversity is a better predictor of subjective well-being benefits than objective measures of biodiversity [17]. While there was no relationship between actual biodiversity (e.g., plant and animal species richness) and psychological well-being, perceived biodiversity by green space users is positively associated with enhanced well-being [18]. Rozario et al. (2024) note that an increase in perceived biodiversity in forests was positively associated with short-term mental health and well-being outcomes [19]. However, this study found no significant relationship between actual biodiversity (specifically tree species richness) and mental well-being. Fisher et al. also note that people are more likely to feel enhanced well-being if they perceive a place as biodiverse, regardless of its actual degree of biodiversity [20].

To amplify the benefits of nature, it is essential to understand which landscape characteristics influence perceived biodiversity. Visual cues constitute a significant factor in determining the perception of biodiversity. Perceived plant species richness is the most widely used indicator for the visual assessment of perceived biodiversity. In other words, it refers to how many plant species people can perceive within green spaces. Since a higher number of plant species is associated with higher visual complexity [21], vegetation features (e.g., height) play a significant role in the ability to perceive biodiversity [22]. Along with these visual cues, auditory cues contribute to perceived biodiversity. Realistic natural sounds (e.g., birdsongs) can increase perceived biodiversity compared to soundless environments [23].

While much scholarly attention has focused on the beneficial effects of biodiversity on mental well-being, the relationships between visual and auditory cues, perceived biodiversity, and restorative effects remain unclear. In particular, the pathway linking visual and auditory cues to perceived biodiversity—and subsequently to restorative benefits—has yet to be fully understood. Illuminating this relationship is crucial, as environmental perception may be as influential as the environment itself. Understanding which landscape design features evoke a sense of biodiversity (i.e., perceived biodiversity) could, in turn, maximize restorative benefits. Perceived biodiversity is positively related to mental well-being and health, and this relationship may be mediated by psychological restoration [24,25]. Visual cues related to structural forest attributes are potentially associated with perceived biodiversity, which is linked to short-term mental health and well-being outcomes [19].

### 1.1. Literature Review

#### 1.1.1. Visual Cues for Perceived Biodiversity and Psychological Response

Vegetation evenness can be assessed by the degree to which abundance is evenly distributed among the different species within a community [26]. Given the same degree of species richness, perceived species richness can be influenced by species evenness—that is, how evenly individuals are distributed among species within an ecosystem. In this respect, species evenness, rather than species richness alone, may play a more significant role in the appreciation of biodiversity [27]. Moreover, vegetation height contributes to visual complexity and is thus related to perceived biodiversity [21], while taller vegetation can increase the perception of naturalness [28]. Plant color may also contribute to the perception of biodiversity. Grose (2012) concludes that detailed information derived from the color diversity of plants supports the understanding of visual biodiversity [29].

The visual presence of plants is a crucial factor in the perception of visual landscape quality, such as aesthetic elements in landscape design [30,31]. Aesthetically pleasing visual stimuli have a potential tension- and stress-reducing effect [32]. From a design perspective, the composition of plants is the main component that determines the overall aesthetic of a landscape [30]. People demonstrate a preference for greater species evenness over lower species evenness [27,33], as well as for visually diverse and heterogeneous landscapes [34,35]. Another important visual component of landscape that affects psychological responses is landscape color [33]. Given that higher hue variation promotes visual fixation [36], plant color impacts the visual characteristics of a landscape and stimulates the human sensory system [37]. Furthermore, plant colors are related to psychological relaxation as they promote positive feelings such as comfort, calmness, and naturalness [38]. Plant height also contributes to the psychological benefits derived from landscapes. Kuper (2017) demonstrated that tree height is positively associated with restorative potential [39]. People are also more likely to prefer taller plants compared to shorter ones [40].

#### 1.1.2. The Auditory–Visual Cues for Perceived Biodiversity and Psychological Responses

Another noteworthy observation in environmental psychology is that the integration of auditory and visual stimuli plays a pivotal role in psychological responses. As the interactions between visual and auditory create an essential influence on perception [41], multisensory information can complement and reinforce them [42,43]. Growing evidence reveals the effect of nature’s multisensory stimuli on cognitive and psychological well-being. Although natural sounds such as birdsong, wind, and water are typically considered more pleasant by people [44,45], birdsong is especially noted as the most effective for mental restoration among these natural sounds [46]. Birdsongs and calls have been associated with restorative effects, including stress recovery and attention restoration [47]. Realistic nature sounds can enhance perceived biodiversity, indicating that people are more likely to perceive greater biodiversity with the presence of both visual and sound stimuli [23]. Further, the combination of auditory and visual stimuli significantly enhances psychological restoration (e.g., stress recovery, emotion, cognitive well-being) and aesthetic preference compared to visual stimulation alone [11,41,42,46].

### 1.2. Research Gaps and Questions

To the best of our knowledge, no studies have verified how perceived biodiversity may act as a mediator between environmental features and restorative benefits while controlling for the level of biodiversity. Our study seeks to fill this gap by constructing hypothetical environments with controlled conditions (e.g., equivalent biodiversity levels across all environmental exposures). This study attempts to answer the following research questions: (1) What visual elements of the landscape (e.g., species evenness, height, color) have an impact on perceived biodiversity and restorative effects? (2) Do auditory cues (e.g., birdsong) continue to affect these perceptions when visual stimuli are also present? (3) Does the perception of biodiversity mediate the relationship between visual–auditory features and restorative effects?

## 2. Methods

### 2.1. Research Design

**Figure 1:**
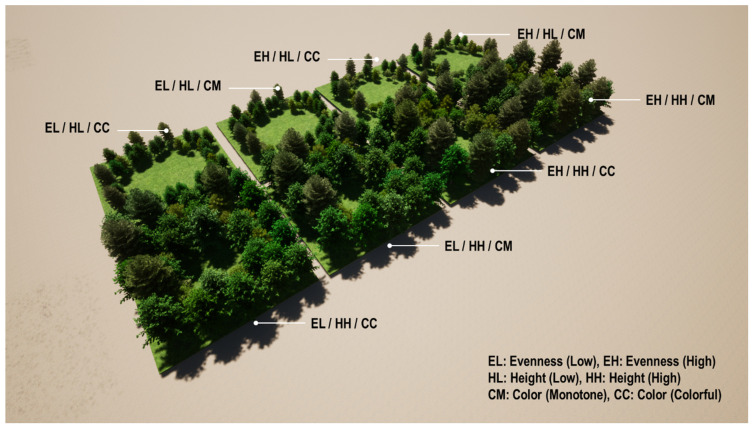
Bird’s eye view of eight different environment settings.

**Figure 2:**
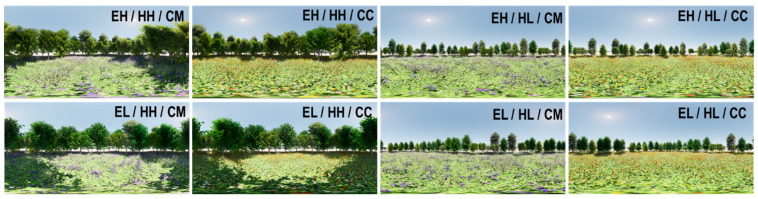
Panorama view of eight environmental conditions at eye level.

This study employed a posttest-only, randomized, 2 × 2 × 2 factorial experiment design to examine the restorative effects of three visual factors and an auditory factor on psychological well-being. A total of eight environments were constructed using combinations of species evenness (two levels: low, high), height (two levels: low, high), and color (two levels: monotone, colorful) (see Table 1). We designed a hypothetical urban green space using the educational version of Twinmotion 2020 (see Figure 1 and Figure 2). Measuring 30 square meters, virtual green spaces are filled with trees and perennials. Non-plant elements that potentially influence participants’ psychology were excluded (e.g., people, animals, man-made objects). The ambiance settings—season, weather, and time—remained default: summer, clear sky with sunlight, and mid-morning time. To control biodiversity, all eight hypothetical environments contained 5 species, including the Katsura tree, Nikko Fir, Japanese white larch, Japanese walnut, and Amur cork tree. Tree species not commonly found in Kansas areas were intentionally selected since the participants’ familiarity with certain tree species may influence the perception of biodiversity (i.e., greater familiarity with certain species may increase recognition ability). Two levels of species evenness were computed using Shannon’s diversity index—Equation (1)—and Shannon’s equitability index—Equation (2) —resulting in low evenness (0.3327) and high evenness (0.4362) (see Table 2 and Table 3), respectively. Two levels of plant height were measured and averaged using data from 40 trees. The average height of the low plant species was 5.03 m, while the average height of the high plant species was 10.09 m (see Supplementary Materials). The height difference was approximately twofold, making it easily perceptible to participants. Two color levels were determined based on the number of flower colors, using three different flower species. This study did not use tree foliage color, as the selected tree species only exhibited yellow fall foliage, offering limited color variation. Moreover, the greenery of trees significantly affects restorative effects; we did not use the foliage color of trees in the experimental setting. The monotone-color environment featured only purple flowers, while the colorful environment included a combination of three colors: yellow, red, and white.

(1) $$H=-\sum_{i=1}^{s}P_{i}\;{ln}_{Pi}$$

(2) $$E_{H}=-H/Ln(S)$$

The height of the trees was controlled by Twinmotion. Two flower color conditions were designed: one using monochromatic colors (i.e., a single color) and the other using a variety of colors (e.g., three different colors). This study employed two sound conditions in each of the eight different environments: with and without sound. We added nature sounds to the simulation using sound effects from Twinmotion, employing birdsongs as the primary natural sound effect in a scene representing a typical urban green space encountered in daily life.

### 2.2. Study Subject

A total of 64 participants recruited from Kansas State University participated in this study. We recruited participants through flyers on the campus. Participants received a USD 10 e-gift card as compensation for participating in this research. The study sample size was calculated using G*power version 3.1.9.7 [48]. This software performs various statistical tests such as *t*-tests, F-tests, χ^2^ tests, and z-tests for determining sample size. The results indicated that a sample size of 25 was required for this study (effect size f = 0.25, α err probability = 0.05, and power (1-β err probability) = 0.8). Our sample size meets this criterion. Among the 64 participants, 29 out of 64 students were male; 48.4% of participants were current undergraduate students and 31.3% were graduate students; 62% of participants were employed and 37.5% were unemployed; 62.5% of participants’ household income ranged from USD 10,000 to USD 29,999. Most participants responded that they were not married. The average age of respondents was 24. This study was approved by the Kansas State University Institutional Review Board (IRB), under approval number IRB-10934.

### 2.3. Research Procedure

**Figure 3:**
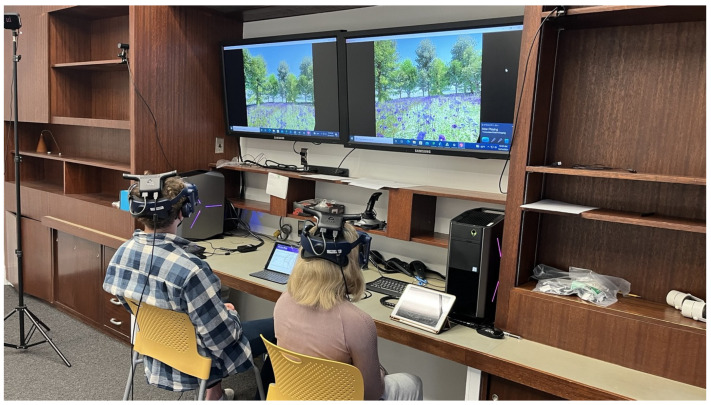
Lab experiment example. Participants wearing HMDs were seated at the desk to complete the experiment.

All participants signed up for a research appointment, with one or two participants in each hour block. The experiment took place in a virtual reality (VR) laboratory in the College of Architecture at Kansas State University. VR was employed in this study because it offers a more immersive user experience and greater environmental realism compared to traditional technologies, such as 2D monitor displays. The laboratory space was a quiet room separated from nearby classrooms, which minimized external influences and disturbances. Two VIVE PRO head-mount displays (HMDs), each with Dual AMOLED 3.5” screens with 2880 × 1600-pixel resolution (1440 × 1600 pixels per eye), were used. The HMD was equipped with a High-Res Soundstage, enabling users to experience immersive sound effects. A VIVE Wireless Adapter was used to connect to desktop computers without a physical cable, so that participants were free to move around (360 degrees) in the simulation wirelessly (see Figure 3).

**Figure 4:**
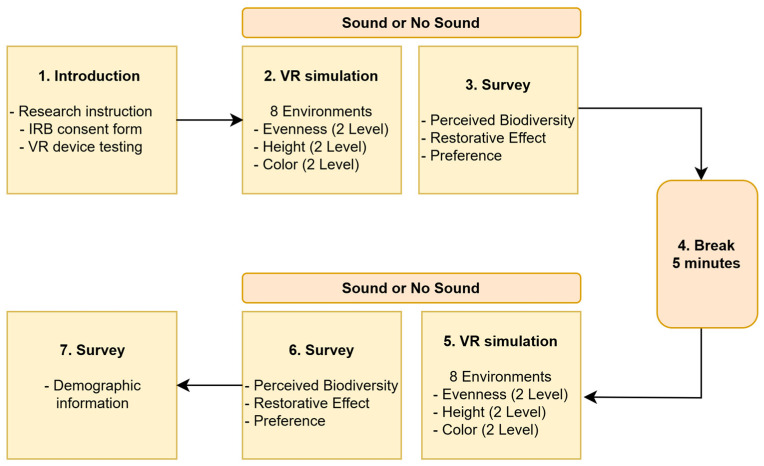
Flow chart of research experiment.

After explaining the research purpose and aims to participants, we asked them to sign an informed consent form (see Figure 4). Participants were then randomly assigned to either no-sound or sound conditions and exposed to eight combinations of environments, varying by evenness (2 levels), height (2 levels), and color (2 levels). Sound and no-sound conditions were used as a blocking factor to examine how visual-only and auditory–visual environments differently influence perception and restorative effects. The sequence of simulated conditions (i.e., sound or no sound) was determined by whichever environment they encountered first, followed by the other settings. The order of simulations was randomized for each participant to ensure counterbalancing and to minimize the effects of order-related confounding variables. Then, participants took a five-minute break between sequences of sound and no-sound sessions to minimize mental fatigue. They were then exposed to 8 combinations of environments under the alternate sound conditions. Within both sound and no-sound conditions, the eight different environments (evenness, height, and color) were randomly presented without any specific sequence. In total, participants were exposed to 16 different environmental conditions, resulting from the combination of sound presence (2 levels) × evenness (2 levels) × height (2 levels) × color (2 levels). Based on previous studies [23,49,50], our experiment adopted a simulation runtime of one minute, which is a minimum duration of environmental exposure to observe changes in psychological response. After each one-minute simulation, participants removed the VR headset and completed questionnaires (e.g., perceived biodiversity, restorative effect, and preference) using the online survey platform Qualtrics. Participants entered their Qualtrics survey responses using an iPad tablet. At the end of the survey, we also inquired about their demographic information (e.g., socioeconomic status). The mean time to conduct this research experiment was 45.18 min ranging from 28.71 min to 106.1 min.

### 2.4. Data and Variables

Measurements of perceived biodiversity were derived from the Biodiversity Experience Index (BEI) [51]. The following question was most relevant to our research settings: “Imagine you are in the projected scene, please select a scale for each item according to your perception (1 = ‘not at all’ to 5 = ‘very much’) regarding (1) Plant richness, (2) Wildness, (3) Variety.” We asked about their preference regarding each simulated environment by asking, “How much do you like this environment? (1 = ‘not at all’ to 5 = ‘very much’)”. Using the short-version Revised Restoration Scale [52], this study assessed perceived restorative outcomes regarding emotional, physiological, cognitive, and behavioral perception via the following question: “Imagine you are in the projected scene; please select a scale for each item according to your perception (1 = ‘totally disagree’ to 9 = ‘totally agree’)”.

### 2.5. Statistical Analysis

This study conducted a repeated-measures ANOVA to assess the mean difference between repeated observations of selected visual actors and evaluate their interaction effects. We also report the partial eta squared (η2) to verify the effect size of each variable. Since all factors had two levels, our study did not consider post hoc tests (e.g., Tukey test). Furthermore, a paired t-test was conducted to compare the effects of sound on selected environmental factors (with sound versus without sound). We calculated effect size using Cohen’s d [53]; the values indicate small (0.2), medium (0.5), and large (0.8) effect sizes. All analyses were performed using the statistical software IBM SPSS 28. Lastly, this study also examined whether perceived biodiversity might act as a mediating factor between perceived biodiversity and mental restoration. Due to the structure of repeated-measure data—each participant was exposed to 16 environments—the nested data violates the assumption of independence. Thus, this study employed a mixed-effect model, which allows for the inclusion of random intercepts to account for individual differences in baseline responses across participants, while estimating fixed effects (e.g., environmental features). To examine mediation effects, Baron and Kenny (1986) mentioned that three separate regression steps need to be tested: (1) regress the mediator on the independent variable (independent variable → mediator); (2) regress the dependent variable on the independent variable (independent variable → dependent variable); (3) regress both the dependent variable and independent variable on the mediator (independent variable + mediator → dependent variable) [54]. The mixed-effect model analysis was conducted using the “lme4” package in R studio 4.2.1 [55].

## 3. Results

### 3.1. The Effects of Visual Intervention on Psychological Responses

**Figure 5:**
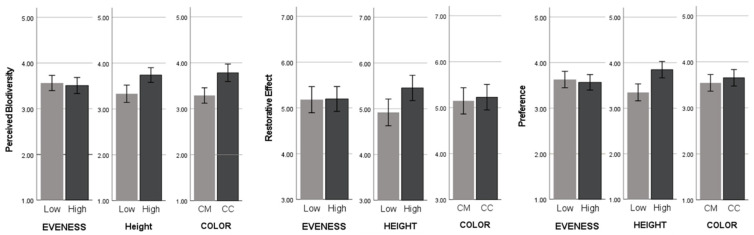
The average scores of perceived biodiversity, restorative effect, and preference.

This study performed a repeated-measures ANOVA analysis to examine the effect of the three main factors—evenness (2 levels), height (2 levels), and color (2 levels)—on psychological responses and their interaction effects on each visual-only environment, along with the corresponding descriptive statistics (see Table 4 and Figure 5).

The results indicated that plant height had a significant main effect on respondents’ perceived biodiversity: F(1,63) = 46.318, *p* < 0.001, η2 = 0.424 (see Table 5). Perceived biodiversity was higher in environments with tall plants in comparison to shorter ones. The effect of plant color on perceived biodiversity was significant: F(1,63) = 51.806, *p* < 0.001, η2 = 0.451. A higher degree of perceived biodiversity was found in more colorful environments. There was a significant interaction effect between plant height and plant color: F (1,63) = 5.437, *p* = 0.023, η2 = 0.079.

The findings revealed that there was a significant effect of plant height on restorative outcomes: F(1,63) = 30.028, *p* < 0.001, η2 = 0.323. The environment with taller plants had a higher degree of restorative outcomes as opposed to shorter plants. There was a significant interaction effect between plant height and plant color: F(1,63) = 6.439, *p* = 0.014, η2 = 0.093.

The results demonstrated that there was a significant influence of plant height on preference: F(1,63) = 51.922, *p* < 0.001, η2 = 0.452. Taller plant environments indicated greater preference effects compared to shorter plant environments. We also found that there was a significant main effect of color on respondents’ preference: F(1,63) = 84.483, *p* < 0.001, η2 = 0.573. The various-color environments were more preferred compared to monotone-color environments. There was a significant interaction effect between plant evenness and height: F(1,63) = 4.492, *p* = 0.038, η2 = 0.067 (see Table 5).

### 3.2. The Effects of the Auditory Intervention on Psychological Responses

**Figure 6:**
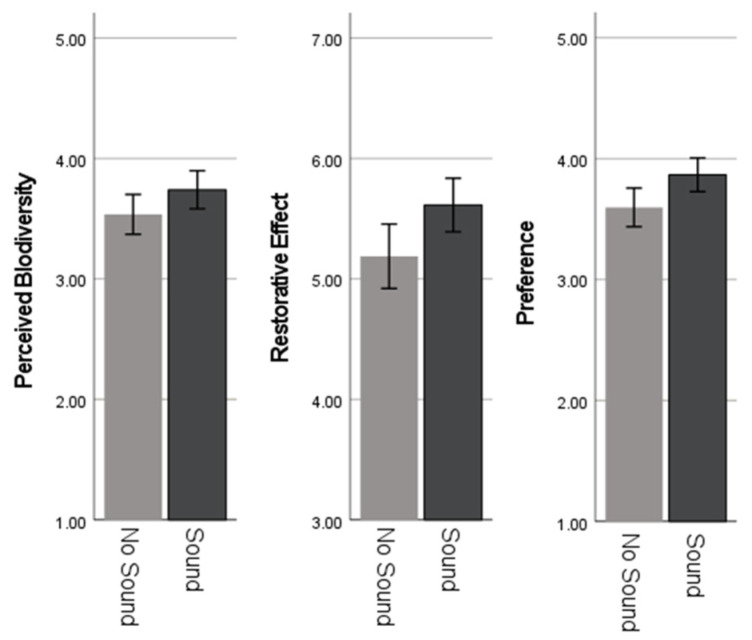
Comparison of sound and no-sound environments on perceived biodiversity, restorative effects, and preference.

The results of the paired *t*-test demonstrated a statistically significant difference between the mean scores of psychological responses in the sound and no-sound environments (see Figure 6). A paired-sample *t*-test showed a t-statistic of −3.810, with df = 63 (*p* < 0.001). The sound environment had a higher mean score in perceived biodiversity (M = 3.740, SD = 0.632) than environments without sound (M = 3.536, SD = 0.662). The effect size, as measured by Cohen’s d, was d = −0.476, indicating a medium effect (see Table 6). A paired-sample *t*-test exhibited a t-statistic of −4.580, with df = 63 (*p* < 0.001). The sound environments had higher mean restorative outcomes (M = 5.614, SD = 0.890) than the environments with no sound (M = 5.188, SD = 1.069). The effect size, as measured by Cohen’s d, was d = −0.572, indicating a medium effect. A paired-sample t-test appeared with a t-statistic of −5.226, with df = 63 (*p* < 0.001). The mean preference score was significantly greater in the sound environments (M = 3.867, SD = 0.557) compared to environments with no sound (M = 3.597, SD = 0.557). The effect size, as measured by Cohen’s d, was d = −0.653, indicating a medium effect (see Table 6).

### 3.3. The Mediating Effects of the Perceived Biodiversity Between Environment Features and Restorative Effects

Linear mixed-effect models were created to examine how visual and auditory features influence perceived biodiversity and restorative effects (see Table 7). Random intercepts for participants were included to account for the repeated-measures design (e.g., 16 repeated environmental exposures). Additionally, we investigated whether perceived biodiversity serves as a potential mediator between auditory–visual stimuli and restorative effects. The intraclass correlation coefficients (ICCs) in all three models indicated substantial variability between participants. The ICCs, ranging from 0.34 to 0.51, were considered to reflect moderate to high variability between participants, supporting the inclusion of random effects. Model 1 tested the impact of visual and auditory features on perceived biodiversity. The results indicated significant positive effects of the sound condition (b = 0.20, *p* < 0.001), height (b = 0.44, *p* < 0.001), and color (b = 0.51, *p* < 0.001). Model 2 verified how visual and auditory features affect restorative effects. The results showed that sound (b = 0.43, *p* < 0.001) and taller vegetation (b = 0.74, *p* < 0.001) significantly improved restorative effects. Model 3 combined visual–auditory features and perceived biodiversity to examine the potential mediation effect. Perceived biodiversity remained a strong predictor of restorative effects (b = 0.74, *p* < 0.001). Sound (b = 0.27, *p* < 0.001), height (b = 0.41, *p* < 0.001), and evenness (b = 0.12, *p* = 0.031) revealed significantly positive effects for restorative effects, while color indicated a negative effect (b = −0.36, *p* < 0.001) (see Table 7).

## 4. Discussion

Our findings revealed that the visual and auditory elements of the landscape impact both cognitive evaluations and psychological responses. In particular, this study provides valuable insight into the distinction between the direct and indirect effects of environmental features. The results of the mixed-effect models illustrate that a multisensory approach to perceived biodiversity and restorative effects is critical for understanding the mechanisms underlying mental restoration in biodiverse environments. Visual elements of the landscape remain important predictors of perceived biodiversity, even when auditory factors are taken into account.

The findings of this study indicated that participants were more likely to perceive higher biodiversity in environments with taller vegetation as compared to environments with shorter vegetation. This result aligns with previous research showing that vegetation height can serve as a reliable indicator of botanical richness [22]. Taller vegetation tends to provide more favorable shelters and diverse ecological conditions for invertebrate communities [56]. Another possible reason is that the height of trees plays a significant role in the perception of naturalness, which is influenced by perceived biodiversity. The public feels a strong sense of naturalness in environments with taller trees [28].

Contrary to our expectations, the results revealed no significant association between species evenness and perceived biodiversity. These findings, supported by previous literature [22], might be explained by the possibility that plant evenness is not as strong an indicator as plant color or height, or because the participants were neither skilled at identifying nor particularly knowledgeable about plant species. It is also possible that participants had difficulty perceiving differences in species evenness within the virtual environment. The experimental setting was not as realistic as an actual site and did not allow participants to freely navigate the space or closely examine the plants.

Participants were more likely to have higher perceived biodiversity when they were in sound environments compared to no-sound environments. This aligns with previous studies showing that natural sound can heighten perceived species richness [23]. One possible assumption is that natural sounds (e.g., birdsongs) create the illusion of biodiversity, leading to the perception of greater ecological diversity at a site. Our finding also provides evidence that natural sound is associated with people’s preferences [42,57,58], as well as restorative effects [46,59,60]. Thus, exposure to nature through multisensory stimuli, including both visual and auditory perception, can promote greater sensory awareness, deepen immersion, and galvanize a stronger sense of nature [45].

Our study found strong evidence that perceived biodiversity partially mediates the relationship between environmental features (both auditory and visual) and restorative effects. Perceived biodiversity consistently demonstrated explanatory power across models. In other words, it consistently emerged as a strong predictor, highlighting its role as a key restorative mechanism. These findings support the idea that restorative effects may be driven not only by the actual characteristics of environments (i.e., actual biodiversity) but also by the extent to which people perceive environments as biodiverse. Considering the mediation effect, higher species evenness and taller vegetation significantly enhance perceived biodiversity, subsequently leading to greater restorative effects. Our findings showed that environments with taller trees were more likely to produce greater restorative effects. A possible explanation is that taller vegetation tends to have a larger canopy, resulting in a greater amount of greenness. As with many previous studies, a dose of greenness is positively related to restorative benefits [2,61,62]. Thus, taller vegetation could lead to a greater restorative effect compared to shorter vegetation.

Contrary to our ANOVA findings, species evenness was positively associated with restorative effects when accounting for the mediation effect of perceived biodiversity. The mixed-effect model indicated a partial mediation pathway linking species evenness, perceived biodiversity, and restorative effects. This suggests that the influence of species evenness may be context-dependent, yet it still contributes to the perceptual mechanisms that support restorative outcomes. Although species evenness alone had minimal direct influence, its interaction with other visual (e.g., vegetation height, color) and auditory cues may enhance landscape complexity, thereby affecting how biodiversity is cognitively constructed. This indirect effect can significantly contribute to the mechanisms underlying restorative outcomes. While color showed a positive effect on perceived biodiversity (Model 1) and in the ANOVA analysis, it had no direct effect on restoration (Model 2). In fact, once perceived biodiversity was included in the model (Model 3), color exhibited a negative effect on restorative outcomes. These contrasting results suggest that while color may enhance the appreciation of biodiversity, a variety of hues and saturation levels could potentially overstimulate visual perception, leading to mental fatigue.

Consistent with previous findings on restorative effects, our study indicated that plant heights were positively associated with visual preference. This result is due to the fact that taller plants are more likely to have a greater degree of greenness at eye level. Existing literature illuminated that vegetation density is positively associated with the level of preference [63,64,65]. Colorful plants had a significant effect on the preferences of participants. Lastly, plant evenness has nothing to do with people’s preferences; we found no evidence that plant evenness was associated with preference. This may be due to the fact that virtually created plants were not sufficiently realistic to enable participants to accurately perceive plant evenness, resulting in no significant discrepancy in preferences among participants.

## 5. Limitations and Implications

Our study had some limitations. First, our experiments were implemented in a virtually created environment. No matter how realistic images can be in VR, the simulated environments do not convey the same realism as in situ and on-site environments. The HMD can potentially cause motion sickness, including dizziness and headaches caused by a sensory mismatch between perceived motion in VR environments and physical stationarity in the real world [66,67,68]. Ideally, a future study could conduct an on-site experiment while controlling all study variables. Second, this research only used subjective measurements of psychological responses, using survey methods. Future studies should consider including objectively measured physiological responses such as heart rate, skin-conductance level, and salivary cortisol, along with subjective measurements. Third, our study did not rigorously control the amount of vegetation, since taller vegetation generally has a higher degree of greenness. However, it is worth investigating how this result varies when controlling for a dose of greenness and vegetation height. Fourth, this research only adopts two levels (low and high) in each factor (evenness, height, and color). To gain more granular insights from this study, we recommend a three-level (or higher) research design to identify linear effects between environmental factors and response variables. Using multiple regression, future research can examine the associations between variables while controlling for socioeconomic status. Although the binary comparison allows for clear differentiation between two distinct environments, our study does not account for how individual perceptions and knowledge may influence participants’ ability to discern these differences. Future research should consider incorporating these individual factors into the study design. Lastly, the individual visual characteristics of each plant species were not considered in this study. However, unique visual features may influence psychological responses and should be explored in future research.

The results of this study indicate that the physical characteristics of plants were associated with perceived species richness, preference, and restorative effects. These findings supplement a larger body of ongoing issues “beyond just green” in landscape design and planning. In practical terms, this study suggests that simply enhancing biodiversity in cities is not sufficient; to maximize the restorative benefits of nature, planners and designers must ensure that biodiversity is visible, perceptible, and emotionally engaging. The findings specifically highlight that green spaces with evenly distributed plant species can help the public perceive biodiversity more clearly, resulting in greater restorative effects compared to spaces dominated by only a few species. Landscape designers should also consider incorporating a diverse color palette in vegetation, rather than relying on monochromatic colors, especially in urban parks where green space is limited in high-density cities. In doing so, people may perceive these environments as more biodiverse and experience stronger restorative benefits. Additionally, taller vegetation may be more effective in enhancing perceived biodiversity and supporting mental well-being. Lastly, by providing livable habitats for bird species, urban green spaces can foster richer natural soundscapes, contributing to more stimulating and multisensory environments.

## 6. Conclusions

This research provides robust evidence on how visual and auditory cues in the landscape influence both cognitive and psychological responses, with notable contrasts between direct and indirect effects. While plant height and natural sound showed consistent direct effects on restorative outcomes, the visual cues such as plant color and evenness influenced restoration indirectly through perceived biodiversity. Mixed-effect modeling demonstrated a partial mediating effect between landscape features and restorative outcomes—an effect not detected in the direct relationships found through ANOVA. Species evenness was not directly related to restorative effects but was indirectly associated through perceived biodiversity. Similarly, although color enhanced perceived biodiversity, it did not have a direct effect on mental restoration and may even produce negative effects under certain conditions. Our findings suggest that the restorative benefits of nature can be achieved not only through the ecological qualities of a landscape but also through how people perceive its biodiversity. Although many more factors contribute to landscape biodiversity, we focused on those that people can easily perceive in their surrounding environments, as such perceptions may be linked to psychological restoration and well-being. This study also offers insights into landscape biodiversity that are relevant to professionals who design landscape spaces. Based on our findings, we strongly recommend that landscape architects and environmental designers incorporate rich, multisensory environments while ensuring that ecological features are clearly perceivable through visual cues.

## Figures and Tables

**Table 1:** Factorial experimental design; evenness (2 levels) × height (2 levels) × color (2 levels) with auditory cues.

Scenario	Visual Cues			Auditory Cues
	Evenness	Height	Color	
Scenario #1	Low	Low	Monotone	No sound
Scenario #2	Low	Low	Colorful	No sound
Scenario #3	Low	High	Monotone	No sound
Scenario #4	Low	High	Colorful	No sound
Scenario #5	High	Low	Monotone	No sound
Scenario #6	High	Low	Colorful	No sound
Scenario #7	High	High	Monotone	No sound
Scenario #8	High	High	Colorful	No sound
Scenario #9	Low	Low	Monotone	Sound
Scenario #10	Low	Low	Colorful	Sound
Scenario #11	Low	High	Monotone	Sound
Scenario #12	Low	High	Colorful	Sound
Scenario #13	High	Low	Monotone	Sound
Scenario #14	High	Low	Colorful	Sound
Scenario #15	High	High	Monotone	Sound
Scenario #16	High	High	Colorful	Sound

**Table 2:** The results of the calculation of species evenness (low).

Name	Num	$P_{i}$	${ln}_{Pi}$	$P_{i}\;{ln}_{Pi}$	Shannon’s Equitability (EH)
Katsura Tree	24	0.6	−0.51083	−0.3065	0.332765
Nikko Fir	4	0.1	−2.30259	−0.23026	
Japanese White Larch	4	0.1	−2.30259	−0.23026	
Japanese Walnut	4	0.1	−2.30259	−0.23026	
Amur Cork Tree	4	0.1	−2.30259	−0.23026	
Total	40	1		1.227529	

**Table 3:** The results of the calculation of species evenness (high).

Name	Num	$P_{i}$	${ln}_{Pi}$	$P_{i}\;{ln}_{Pi}$	Shannon’s Equitability (EH)
Katsura Tree	8	0.2	−1.60944	−0.32189	0.436295
Nikko Fir	8	0.2	−1.60944	−0.32189	
Japanese White Larch	8	0.2	−1.60944	−0.32189	
Japanese Walnut	8	0.2	−1.60944	−0.32189	
Amur Cork Tree	8	0.2	−1.60944	−0.32189	
Total	40	1		1.609438	

**Table 4:** Descriptive statistics for perceived biodiversity, restorative effect, and preference by 8 different environmental settings.

	Evenness		Height		Color	
	Low	High	Low	High	Monotone	Colorful
Perceived Biodiversity	3.564 (0.084)	3.509 (0.088)	3.332 (0.095)	3.741 (0.081)	3.289 (0.084)	3.784 (0.095)
Restorative Effect	5.181 (0.143)	5.197 (0.136)	4.921 (0.146)	5.456 (0.138)	5.149 (0.143)	5.229 (0.139)
Preference	3.629 (0.090)	3.566 (0.085)	3.348 (0.093)	3.848 (0.090)	3.543 (0.091)	3.652 (0.090)

Notes: values are mean (min: 1; max: 9), with standard deviations inside parentheses.

**Table 5:** Results of the repeated-measures ANOVA for perceived biodiversity, restorative effect, and preference.

Variables	Interaction	Sum of Squares	df	Mean Squares	F	*p*-Value	Partial Eta Squared
Perceived Biodiversity	Evenness	0.383	1	0.383	1.404	0.240	0.022
	Height	21.397	1	21.397	46.318	<0.001 ***	0.424
	Color	31.337	1	31.337	51.806	<0.001 ***	0.451
	Evenness × Height	1.125	1	1.125	3.679	0.060	0.055
	Evenness × Color	0.195	1	0.195	1.188	0.280	0.019
	Height × Color	0.834	1	0.834	5.437	0.023 **	0.079
	Evenness × Height × Color	0.087	1	0.087	0.650	0.423	0.010
Restorative Effect	Evenness	0.033	1	0.033	0.040	0.841	0.001
	Height	36.591	1	36.591	30.028	<0.001 ***	0.323
	Color	0.811	1	0.811	0.807	0.372	0.013
	Evenness × Height	1.953	1	1.953	2.796	0.099	0.042
	Evenness × Color	0.935	1	0.935	2.415	0.125	0.037
	Height × Color	2.876	1	2.876	6.439	0.014 **	0.093
	Evenness × Height × Color	0.007	1	0.007	0.015	0.904	0.000
Preference	Evenness	0.587	1	0.587	1.357	0.248	0.021
	Height	28.438	1	28.438	51.922	<0.001 ***	0.452
	Color	35.420	1	35.420	84.483	<0.001 ***	0.573
	Evenness × Height	1.253	1	1.253	4.492	0.038 **	0.067
	Evenness × Color	0.313	1	0.313	2.098	0.152	0.032
	Height × Color	1.253	1	1.253	7.791	0.007 ***	0.110
	Evenness × Height × Color	0.008	1	0.008	0.056	0.813	0.001

** Significant at the 5% level. *** Significant at the 1% level.

**Table 6:** Paired *t*-test results for psychological responses in environments with and without sound.

					95% Confidence Interval of the Difference					
		Mean	SD	Std. Error Mean	Lower	Upper	t	df	*p*-Value	Cohen’s d
Perceived Biodiversity	No Sound	3.536	0.662	0.082	−0.311	−0.097	−3.810	63	<0.001 ***	−0.476
	Sound	3.740	0.632	0.079						
Restorative Effect	No Sound	5.188	1.069	0.133	−0.610	−0.239	−4.580	63	<0.001 ***	−0.572
	Sound	5.614	0.890	0.111						
Preference	No Sound	3.597	0.640	0.080	−0.372	−0.166	−5.226	63	<0.001 ***	−0.653
	Sound	3.867	0.557	0.069						

*** Significant at the 1% level. SD is the standard deviation.

**Table 7:** Results of mixed-effect models.

	Model 1 Perceived Biodiversity			Model 2 Restorative Effects			Model 3 Restorative Effects		
Predictors	Est	CI	*p*	Est	CI	*p*	Est	CI	*p*
(Intercept)	3.09	2.93–3.26	<0.001 ***	4.77	4.52–5.03	<0.001 ***	2.48	2.14–2.83	<0.001 ***
Sound	0.20	0.13–0.28	<0.001 ***	0.43	0.31–0.54	<0.001 ***	0.27	0.16–0.38	<0.001 ***
Evenness	−0.06	−0.13–0.01	0.090	0.07	−0.04–0.19	0.222	0.12	0.01–0.23	0.031 **
Height	0.44	0.37–0.51	<0.001 ***	0.74	0.62–0.86	<0.001 ***	0.41	0.30–0.53	<0.001 ***
Color	0.51	0.44–0.58	<0.001 ***	0.02	−0.10–0.14	0.760	−0.36	−0.48–−0.24	<0.001 ***
Perceived Biodiversity							0.74	0.65–0.83	<0.001 ***
σ^2^	0.33			0.94			0.78		
τ_00_	0.35			0.77			0.40		
ICC	0.51			0.45			0.34		
N	64			64			64		
Observations	1024			1024			1024		
Marginal R^2^/Conditional R^2^	0.154/0.589			0.096/0.503			0.320/0.548		

** Significant at the 5% level. *** Significant at the 1% level.

## Data Availability

The datasets presented in this article are not readily available in order to protect participant privacy and comply with institutional ethical guidelines.

## Supplementary Materials

The following supporting information can be downloaded at https://www.mdpi.com/article/10.3390/ijerph22081267/s1. Table S1: Plant specification.
