# Supplementary material for: Linking Visual–Auditory Cues to Restoration: The Mediating Role of Perceived Biodiversity
Source: Int J Environ Res Public Health. 2025 Aug 13;22(8):1267. doi: 10.3390/ijerph22081267 (PMC12386299; doi:10.3390/ijerph22081267)
Supplement: Supplementary file 1 [file ijerph-22-01267-s001.zip › ijerph-3752541-supplementary.pdf]

Supplementary documents

Table S1. Plant specification.

Plant Height (Low)

Name	Tree num 1	Tree num 2	Tree num 3	Tree num 4	Tree num 5	Tree num 6	Tree num 7	Tree num 8	AVG
Katsura Tree	13.3	12.6	10.8	11.1	12.4	11.3	11.1	12.3	11.8625
Nikko Fir	15.5	13.5	13.9	15.5	14.2	15.3	16	16.1	15
Japanese White Larch	9.7	11.1	9.4	10.9	10.7	9.4	9.2	9.6	10
Japanese Walnut	7.2	7.6	7.6	6.8	7.6	7.7	7.1	6.8	7.3
Amur Cork Tree	6.9	5.8	5.9	5.9	6.9	6.5	6.5	5.9	6.2875
Average									10.09

Units: meters

Plant Height (High)

Name	Tree num 1	Tree num 2	Tree num 3	Tree num 4	Tree num 5	Tree num 6	Tree num 7	Tree num 8	AVG
Katsura Tree	6.7	6.3	5.4	5.5	6.2	5.6	5.6	6.1	5.925
Nikko Fir	7.7	6.7	7	7.7	7.1	7.6	8	8	7.475
Japanese White Larch	4.8	5.5	4.7	5.5	5.3	4.7	4	4.8	4.9125
Japanese Walnut	3.6	3.8	3.8	3.4	3.8	3.8	3.5	3.4	3.6375
Amur Cork Tree	3.4	2.9	3	3	3.5	3.3	3.3	3	3.175
Average									5.025

Units: meters
